# Differential Effects of PERK and IRE1α Silencing on Expression of Apoptosis and Autophagy Markers in T-Lymphoblastic Leukemia MOLT-3 Cells

**DOI:** 10.3390/ijms27156588

**Published:** 2026-07-24

**Authors:** Ekaterina Sergeevna Prokopenko, Tatyana Vladimirovna Sokolova, Olga Vladimirovna Nadei, Anastasia Dmitrievna Trubnikova, Natalia Ivanovna Agalakova

**Affiliations:** Sechenov Institute of Evolutionary Physiology and Biochemistry, Russian Academy of Sciences, 44 Thorez Avenue, Saint-Petersburg 194223, Russia

**Keywords:** T-lymphoblastic leukemia MOLT-3 cells, RNA interference, PERK, IRE1α, apoptosis, autophagy

## Abstract

Cancer cells are able to survive under conditions of high endoplasmic reticulum (ER) stress by activating the adaptive unfolded protein response (UPR), which is closely linked with autophagy. On the other hand, excessive and prolonged ER stress leads to apoptosis. However, the relationships between different UPR branches and apoptosis or autophagy vary in cancer cells of different origins and depend on the extent and nature of the stress signal. This study was designed to establish the role of ER stress sensors protein kinase RNA-like endoplasmic reticulum kinase (PERK) and inositol-requiring enzyme 1 (IRE1α) in apoptosis or autophagy signaling in T-lymphoblastic leukemia MOLT-3 cells via the RNA interference method. The cells were transfected with small interfering RNAs (si-PERK, si-IRE1α, or si-Cont) for 6 h and further cultured under normal conditions for 72 h to provide an insight into chronic effects of the gene silencing. The expression of apoptosis and autophagy effectors at the mRNA and protein levels was compared using RT-PCR and Western blot assays, respectively. Transfection of the cells with PERK siRNA led to a significant decrease in PERK protein and gene expression, and decreased phosphorylation of its downstream effector eukaryotic initiation factor 2α (eIF2α). PERK silencing was accompanied by activation of apoptosis-related genes and proteins—BCL2-associated X (Bax), caspase-3, C/EBP homologous protein (CHOP), while the levels of autophagy markers (Unc-51 like autophagy activating kinase 1 (ULK1), Beclin-1, and microtubule-associated proteins 1A/1B light chain 3 (LC3A/B)) remained stable. In contrast, treatment of the cells with si-IRE1α reduced the content of IRE1α, X-box-binding protein 1 (sXBP1), and glucose-regulated protein 78 (GRP78) proteins, but increased *ERN1* gene expression. IRE1α RNA interference did not affect the levels of the pro-apoptotic marker Bax, but suppressed caspase-3, CHOP, c-Jun N-terminal kinase (JNK), and autophagy signaling molecules (ULK1, Beclin-1, LC3A/B) at both the transcriptional and translational levels. These results indicate that the PERK pathway is an important contributor to the survival of MOLT-3 cells under basal ER stress, while PERK depletion compromises the resistance of cells to UPR-mediated apoptosis. The IRE1α UPR branch is directly linked with autophagy-dependent signaling, although IRE1α knockdown exerted a more complicated influence on the cells, probably via activation of multiple pro-death and compensatory pro-survival regulatory mechanisms.

## 1. Introduction

A major challenge in clinical oncology is the emergence of tumors resistant to cytotoxic drugs due to genetic mutations or genomic variations [1,2]. The causes and mechanisms of chemoresistance are diverse, but they can be associated with a pathological level of endoplasmic reticulum (ER) stress caused by accumulation of misfolded proteins in the ER lumen [3,4,5]. To maintain the ER capacity for proper protein folding, cells activate several defense mechanisms collectively known as the unfolded protein response (UPR). This regulatory network comprises three signaling pathways initiated by protein kinase RNA-like endoplasmic reticulum kinase (PERK), inositol-requiring enzyme 1 (IRE1), and activating transcription factor 6 (ATF6). Upon dissociation from the ER chaperone, glucose-regulated protein 78 (GRP78), dimerized and autophosphorylated PERK phosphorylates eukaryotic initiation factor 2α (eIF2α), thereby inhibiting global protein synthesis to prevent further accumulation of misfolded proteins while allowing the translation of stress-response genes such as activating transcription factor 4 (ATF4). ATF4, in turn, regulates the transcription of adaptive UPR genes as well as genes involved in apoptosis and autophagy, including C/EBP homologous protein (CHOP) and growth arrest and DNA damage-inducible protein 34 (GADD34). IRE1α, which also undergoes autophosphorylation and dimerization, splices XBP1 (X-box-binding protein 1) mRNA to generate the transcription factor sXBP1. sXBP1 helps restore ER homeostasis by selectively activating genes encoding ER chaperones and components of the ERAD (ER-associated degradation) pathway, while also degrading mRNAs of misfolded ER proteins via RIDD (regulated IRE1-dependent decay). Stimulated ATF6 (pATF6α) translocates to the nucleus, where it triggers the transcription of *XBP1*, UPR-related genes (including *GRP78*), and genes encoding ERAD proteins. By reducing global protein synthesis, promoting the degradation of misfolded proteins, and activating survival genes, these pathways reestablish ER homeostasis and restore normal protein-folding capacity. The ability of cancer cells to effectively sustain the UPR in order to overcome mild, prolonged ER stress ensures their survival, growth, invasion, and metastasis. Moreover, in malignancies of various types, this capability determines their resistance to chemotherapy and radiotherapy [3,4,5].

On the other hand, ER stress is closely linked with autophagy—the catabolic process used by cells for the selective disposal of unnecessary or damaged proteins, macromolecules, and organelles in lysosomes [6]. It serves as an alternative energy source to support cell proliferation, tumor growth, metastasis, adaptation to adverse conditions, and resistance to cytotoxic compounds. Continuous activation of autophagy in tumor cells under ER stress often plays a pro-survival role by establishing a new homeostatic state [7]. In contrast, when ER stress becomes extremely severe or prolonged, it triggers apoptosis, which shares several signaling pathways with autophagy and primarily occurs through the mitochondrial pathway [8,9,10,11]. The dominance of apoptosis or autophagy induced by ER stress depends on the type and degree of the stimuli. However, the role of each UPR signaling pathway in executing adaptive or terminal signals is known to vary depending on the cell type and the nature and level of the stress stimuli.

The present study was designed to partially clarify the relationships between UPR, autophagy, and apoptotic signaling in T-lymphoblastic leukemia MOLT-3 cells. This type of leukemia accounts for 15–20% of all acute lymphoblastic leukemia cases and is characterized by uncontrolled division of immature lymphoid cells (lymphoblasts), affecting the hematopoietic system (bone marrow, lymph nodes, spleen) and the central nervous system in the case of recurrence after chemotherapy [12,13]. As in solid cancers, cells of hematopoietic tumors activate UPR to reestablish ER homeostasis. Under hypoxic conditions in the bone marrow niches, high levels of ER stress and UPR promote proliferation, survival, and multipotency of leukemia cells, as well as determine disease progression and chemoresistance [14,15].

The work focused on the PERK and IRE1α signaling pathways. To establish which of these UPR branches contributes to survival of leukemia cells, and which is associated with their death, the changes in the expression of apoptosis and autophagy effectors were analyzed using small interfering RNA (siRNA). RNA interference, a process of post-transcriptional degradation of specific mRNAs that disrupts their translation, is an important approach to study cellular signaling cascades. Moreover, it is actively used as an experimental approach in anti-cancer therapy which can destroy the mRNAs of oncogenes and the proteins ensuring tumor survival, thus increasing the effectiveness of standard cytotoxic drugs [16,17]. However, recent findings suggest that PERK and IRE1α pathways are not parallel but rather function as an intertwined network. Both cascades were found to be essential for the transcriptional regulation of autophagy genes, whereas IRE1α, but not PERK, is strictly required for the induction of apoptosis under ER stress [18,19,20]. Therefore, the responses of the cells to silencing of individual UPR sensors might vary and influence each other. The goal of the present work was to compare the effect of RNA interference targeting PERK and IRE1α on the expression of several apoptosis- and autophagy-associated proteins in MOLT-3 cells. The protein and/or gene expression of PERK and IRE1α, their downstream effectors (p-eIF2α, sXBP1), the ER chaperone GRP78, canonical (B-cell lymphoma 2 (Bcl-2), BCL2-associated X (Bax), caspase-3) and UPR-related (CHOP, c-Jun N-terminal kinase (JNK)) apoptotic markers, and autophagy effectors (Unc-51 like autophagy activating kinase 1 (ULK1), Beclin-1, microtubule-associated proteins 1A/1B light chain 3 (LC3)) was analyzed. Importantly, the cells in our study were cultured under basal conditions, without any chemical induction of ER stress.

## 2. Results

### 2.1. Effect of PERK and IRE1α RNA Interference on Viability of MOLT-3 Cells

**Figure 1:**
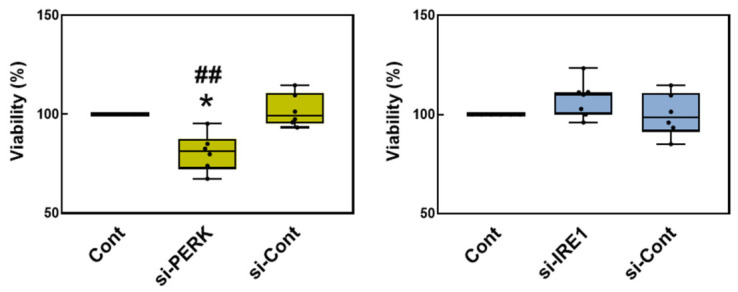
Viability of MOLT-3 cells treated with si-PERK, si-IRE1α and scrambled si-RNAs for 6 h. The viability of cells cultured under control conditions was taken as 100%. Cont—control without transfection, si-PERK—RNA interference of *EIF2AK3* gene, si-IRE1—RNA interference of *ERN1* gene, si-Cont—transfection with control RNA. * *p* < 0.05 in comparison to control; ## *p* < 0.01 compared to transfection with control siRNAs (n = 6, Kruskal–Wallis criterion for multiple comparisons with Dunn test).

The viability of MOLT-3 cells following PERK RNA interference (silencing of *EIF2AK3* gene) decreased by 20% on average compared to that of both the untreated cells and the cells incubated with scrambled si-RNAs (Figure 1). In contrast, the viability of cells cultured with si-IRE1α (silencing of *ERN1* gene) was comparable to both control cells and the cells transfected with control siRNAs.

### 2.2. RNA Interference of PERK in MOLT-3 Cells 

#### 2.2.1. PERK Silencing Suppresses PERK-eIF2α but Not IRE1α-XBP1 Signaling

To confirm the efficiency and specificity of PERK silencing in MOLT-3 cells, the expression levels of PERK and its downstream target eIF2α, the second ER stress sensor IRE1α and its effector sXBP1, as well as the ER chaperone GRP78, have been analyzed.

**Figure 2:**
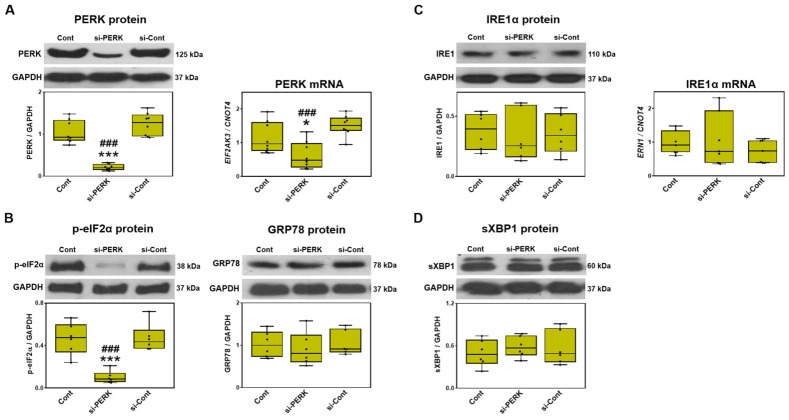
The efficiency and specificity of PERK silencing in MOLT-3 cells. (**A**) Relative levels of PERK protein and mRNA following 6 h PERK RNA interference. (**B**) Protein content of phospho-eIF2α and GRP78. (**C**) Protein and mRNA expression of IRE1α in cells after 6 h si-PERK treatment. (**D**) Cellular level of spliced form of transcription factor XBP1. Shown are typical immunoblots for one sample from each group, average values of protein expression normalized to the optical density of GAPDH (n = 6, one-way ANOVA for multiple comparisons followed by Dunnett post hoc test), and average values of gene expression normalized to reference gene CNOT4 (n = 8, Kruskal–Wallis criterion with Dunn test). Cont—control without transfection, si-PERK—RNA interference of *EIF2AK3* gene, si-Cont—transfection with control RNA. * *p* < 0.05, *** *p* < 0.001 compared to control; ### *p* < 0.001 compared to transfection with control siRNAs.

As expected, treatment of MOLT-3 cells with si-PERK for 6 h resulted in a pronounced downregulation of *EIF2AK3* gene and strongly suppressed the level of PERK protein compared to both untreated cells and the cells transfected with control siRNA (Figure 2A). Accordingly, the cellular content of the active phosphorylated form of eIF2α (p-eIF2α) declined (Figure 2B). However, the protein level of ER chaperone GRP78 remained stable. At the same time, PERK silencing did not have any significant impact on the gene and protein expression of IRE1α (Figure 2C), and on the level of active spliced form of transcription factor XBP1 (sXBP1) (Figure 2D). These results indicate that treatment of MOLT-3 cells with si-PERK specifically and effectively suppressed PERK, but not IRE1α signaling.

#### 2.2.2. PERK Knockdown Enhances the Expression of Apoptotic Proteins in MOLT-3 Cells

**Figure 3:**
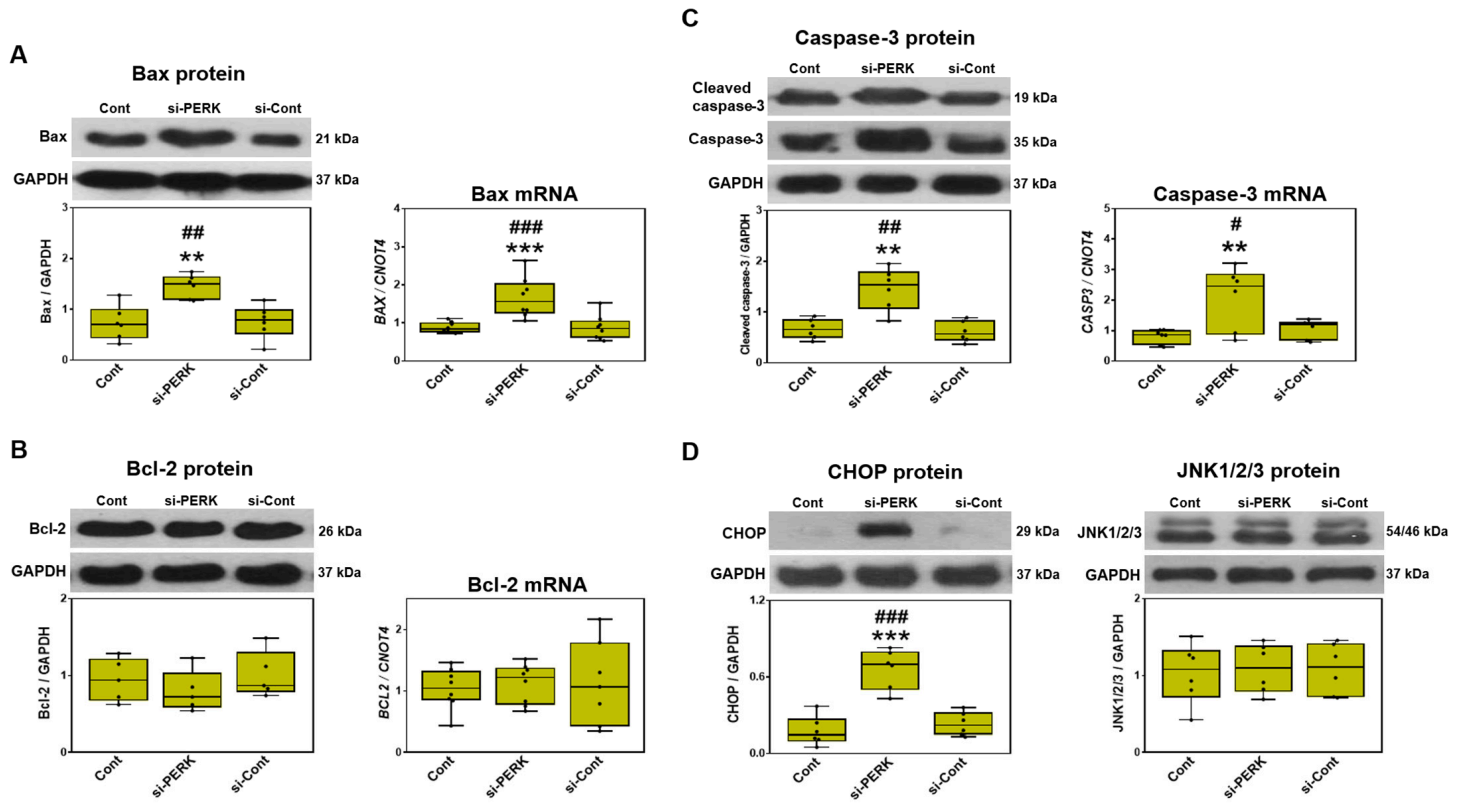
Expression of apoptosis-related proteins and genes in MOLT-3 cells after RNA interference of *EIF2AK3* gene. (**A**) Expression of Bax at translational and transcriptional levels. (**B**) Bcl-2 protein and gene expression. (**C**) Relative expression levels of *CASP3* gene, total and cleaved forms of caspase-3. (**D**) Protein content of transcription factor CHOP and protein kinase JNK1/2/3. Shown are typical immunoblots for one sample from each group, average data for the levels of studied proteins (n = 6, one-way ANOVA, Dunnett test), and average values for gene expression (n = 8, Kruskal–Wallis criterion, Dunn test). Cont—control without transfection, si-PERK—RNA interference of the *EIF2AK3* gene, si-Cont—transfection with control siRNA. ** *p* < 0.01, *** *p* < 0.001 compared to control; # *p* < 0.05, ## *p* < 0.01, ### *p* < 0.001 compared to control transfection.

We next examined how PERK RNA interference influences the expression of mitochondria-associated and ER stress-related apoptotic proteins in MOLT-3 cells. Downregulation of the *EIF2AK3* gene led to activation of the well-established pro-apoptotic effector Bax at both the transcriptional and translational levels (Figure 3A). At the same time, the expression of the anti-apoptotic marker Bcl-2 did not differ from that observed in cells treated with si-Cont or cultured under control conditions (Figure 3B). Such upregulation of Bax, coupled with stable Bcl-2 expression, indicates an increase in the Bax/Bcl-2 ratio, which is typical of mitochondrial apoptotic pathway activation (*p* < 0.01 for both Cont vs. si-PERK and si-Cont vs. si-PERK). Similar to Bax, si-PERK transfection increased the expression of the *CASP3* gene and both forms of the caspase-3—the mature 35 kDa protein and its active 19 kDa cleaved form (Figure 3C). Moreover, the protein level of CHOP, a well-known UPR-associated apoptotic transcription factor, was increased in PERK-knockdown cells, while the level of the protein kinase JNK remained unchanged (Figure 3D). These findings suggest that PERK activity is closely linked to the survival of MOLT-3 cells under basal ER stress, whereas its depletion is associated with UPR-driven cell death. 

#### 2.2.3. Gene and Protein Expression of Autophagy Markers After Silencing EIF2AK3 Gene in MOLT-3 Cells

**Figure 4:**
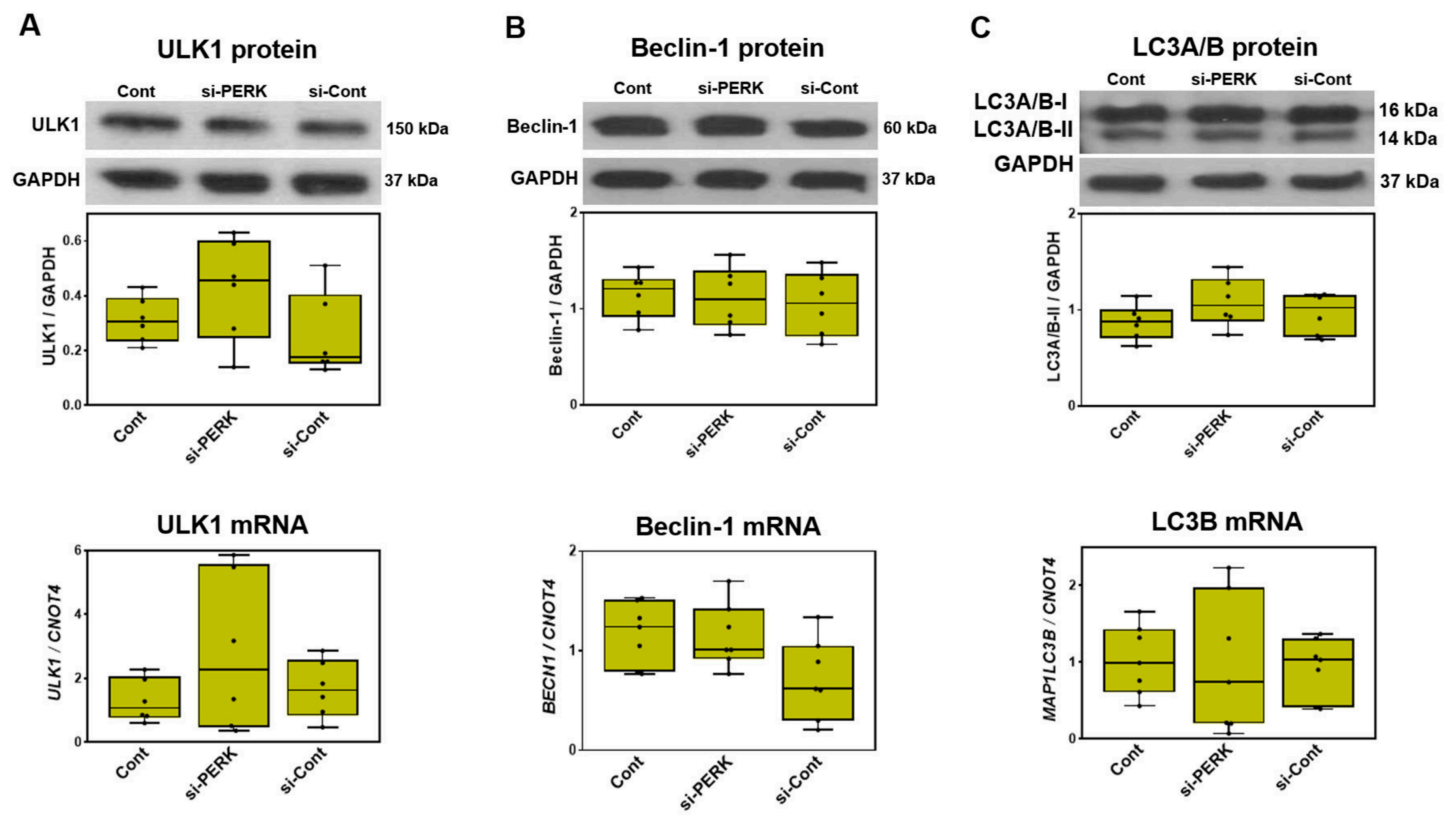
PERK silencing did not affect the expression of autophagy proteins and corresponding genes in MOLT-3 cells. (**A**) Relative expression of ULK1 protein and gene. (**B**) Beclin-1 protein and mRNA levels. (**C**) LC3A/B expression at translational and transcriptional levels. Cont—control without transfection, si-PERK—RNA interference of the *EIF2AK3* gene, si-Cont—transfection with control RNA.

The suppression of *EIF2AK3* gene activity in MOLT-3 cells did not have any significant impact on the expression of key functional proteins and genes of the main stages of autophagy—ULK1, which initiates the autophagy process, Beclin-1, which triggers phagophore maturation, and the marker of autophagosomes/autophagolysosomes LC3A/B (Figure 4).

### 2.3. IRE1α Silencing in MOLT-3 Cells

#### 2.3.1. RNA Interference of IRE1α Influences Both IRE1α and PERK Pathways

**Figure 5:**
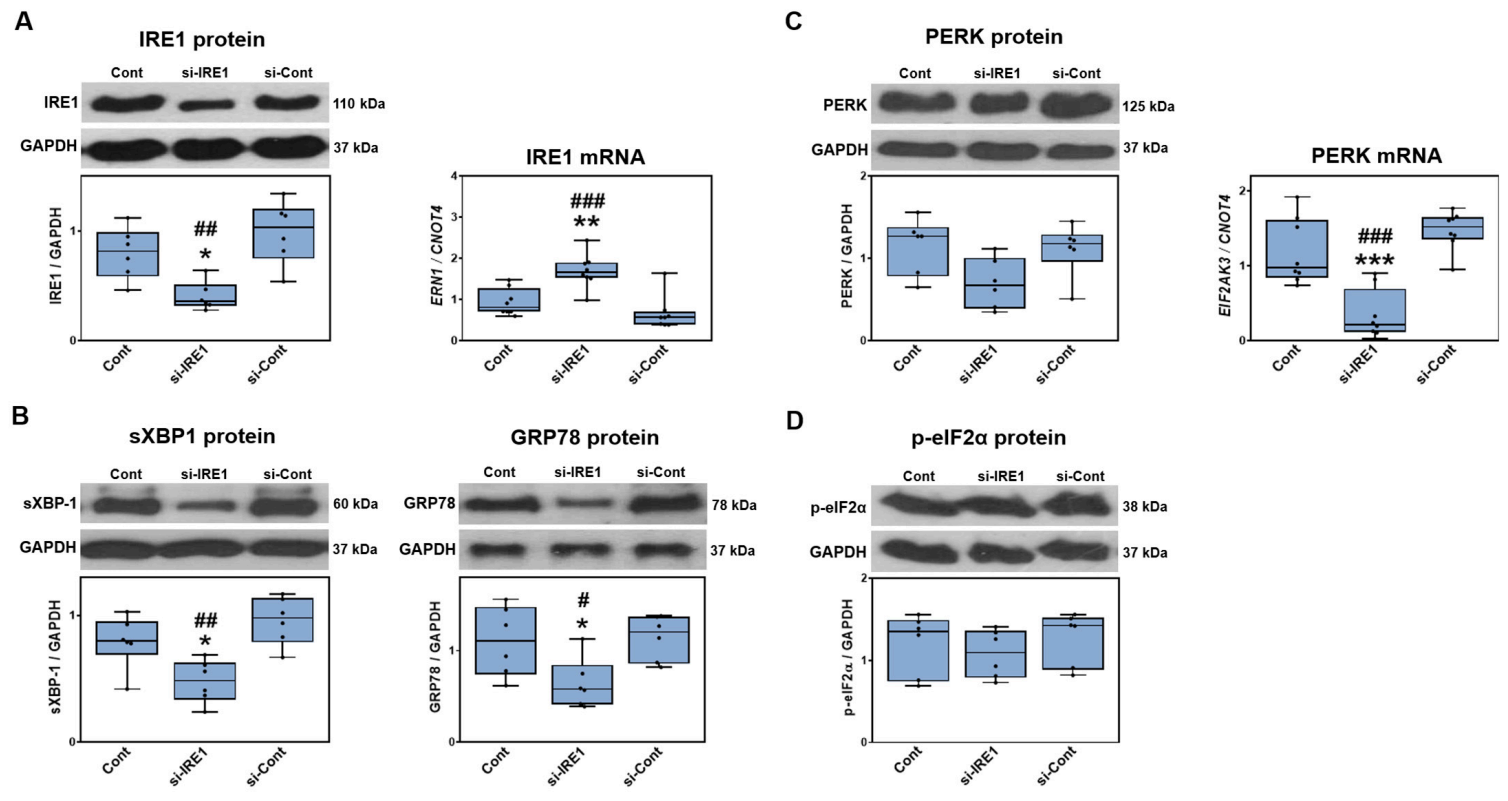
IRE1α silencing in MOLT-3 cells inhibits the translation of IRE1α protein, but increases the expression of *ERN1* gene, and simultaneously suppresses *EIF2AK3* gene. (**A**) Relative levels of IRE1α protein and mRNA in MOLT-3 cells after IRE1α RNA interference. (**B**) Protein content of transcription factor sXBP1 and ER chaperone GRP78 following IRE1α silencing. (**C**) The effect of si-IRE1 on expression of PERK protein and mRNA content. (**D**) Cellular level of phopsho-eIF2α protein. Shown are typical immunoblots for one sample from each group, average values of protein expression normalized to the optical density of GAPDH (n = 6, one-way ANOVA for multiple comparisons followed by Dunnett post hoc test), and average values of gene expression normalized to reference gene CNOT4 (n = 8, Kruskal–Wallis criterion for multiple comparisons with Dunn test). Cont—control without transfection, si-IRE1—RNA interference of *ERN1* gene, si-Cont—transfection with control RNA. * *p* < 0.05, ** *p* < 0.01, *** *p* < 0.001 compared to control; # *p* < 0.05, ## *p* < 0.01, ### *p* < 0.001 compared to transfection with control siRNAs.

Transfection of MOLT-3 cells with IRE1α siRNA yielded unexpected results. In contrast to PERK siRNA, treatment with si-IRE1α for 6 h led to a moderate decrease in IRE1α protein content (Figure 5A). Moreover, IRE1α RNA interference increased expression of the *ERN1* gene (Figure 5A), which might at least partially explain the relatively modest reduction in its protein level following knockdown and suggests a compensatory activation of transcription. Nevertheless, IRE1α loss was accompanied by downregulation of its effector sXBP1 and the ER chaperone GRP78 (Figure 5B). Interestingly, IRE1α silencing also suppressed expression of the *EIF2AK3* gene (Figure 5C), although PERK protein levels remained unchanged (Figure 5C). Taken together, these results likely suggest the existence of mechanisms connecting the two UPR branches.

#### 2.3.2. The Effect of IRE1α RNA Interference on Expression of Apoptosis-Associated Proteins in MOLT-3 Cells

**Figure 6:**
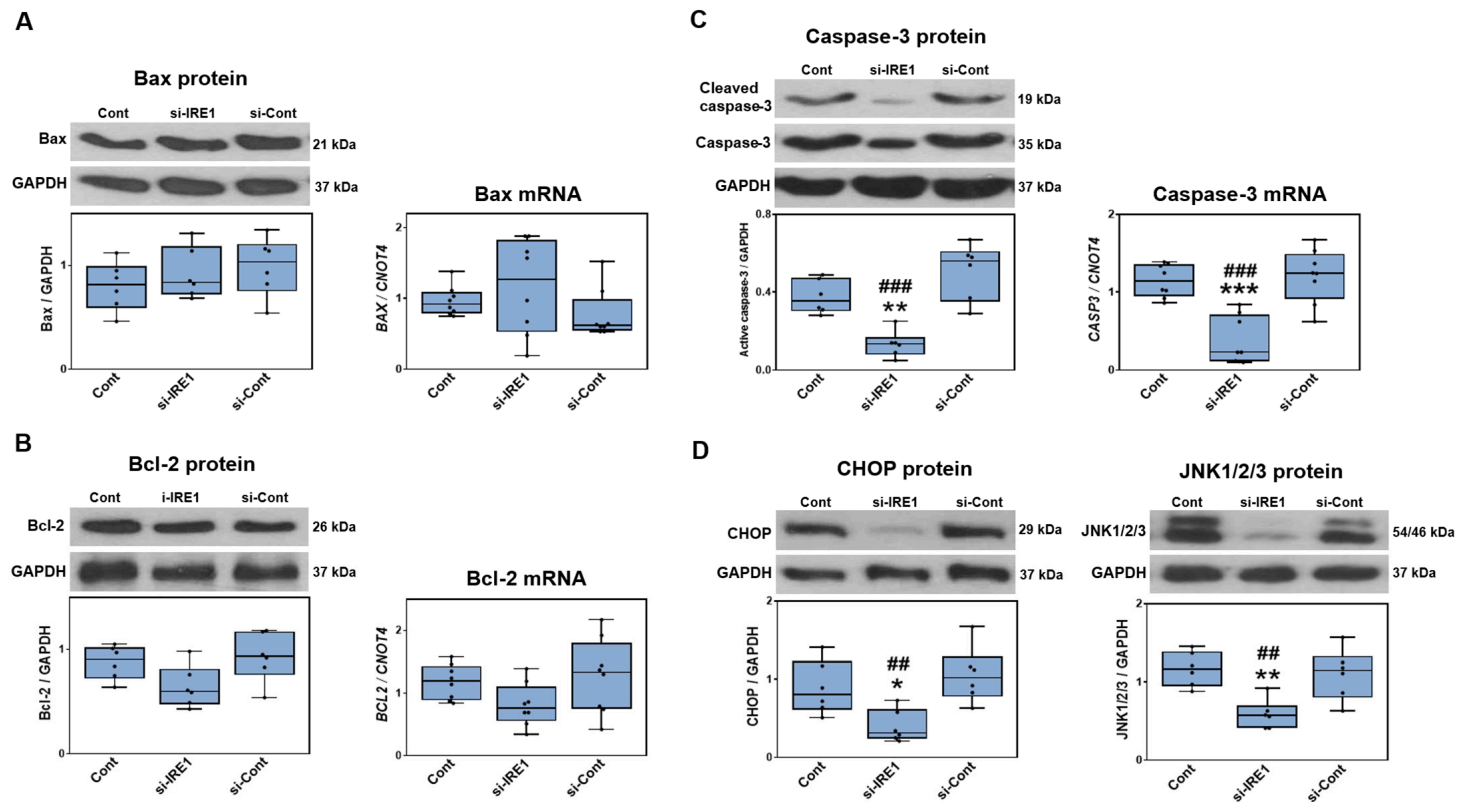
The effect of RNA interference of *ERN1* gene on the expression of apoptosis-related proteins in MOLT-3 cells. (**A**) Expression of Bax at the translational and transcriptional levels. (**B**) Bcl-2 protein and gene expression. (**C**) Relative expression levels of *CASP3* gene, total and cleaved forms of caspase-3. (**D**) Cellular levels of transcription factor CHOP and protein kinase JNK1/2/3. Shown are typical immunoblots for one sample from each group, average data for the levels of studied proteins (n = 6, one-way ANOVA, Dunnett test), and average values for gene expression (n = 8, Kruskal–Wallis criterion, Dunn test). Cont—control without transfection, si-IRE1—RNA interference of *ERN1* gene, si-Cont—transfection with control RNA. * *p* < 0.05, ** *p* < 0.01, *** *p* < 0.001 compared to control; ## *p* < 0.01, ### *p* < 0.001 compared to the control transfection.

Knockdown of *ERN1* gene did not alter the expression of the pro-apoptotic protein Bax (Figure 6A) or the anti-apoptotic effector Bcl-2 (Figure 6B) at either the transcriptional or translational level. However, treatment with si-IRE1 decreased the cellular levels of both the total and active forms of caspase-3, as well as its gene expression (Figure 6C). Transfection with si-IRE1α also suppressed the JNK1/2/3 protein kinases and the UPR-associated pro-apoptotic regulator CHOP (Figure 6D). These data suggest that the IRE1α pathway positively regulates UPR-associated, but not mitochondrial, apoptosis in MOLT-3 cells under basal ER stress.

#### 2.3.3. RNA Interference of IRE1α Inhibits the Expression of Autophagy Genes and Proteins

**Figure 7:**
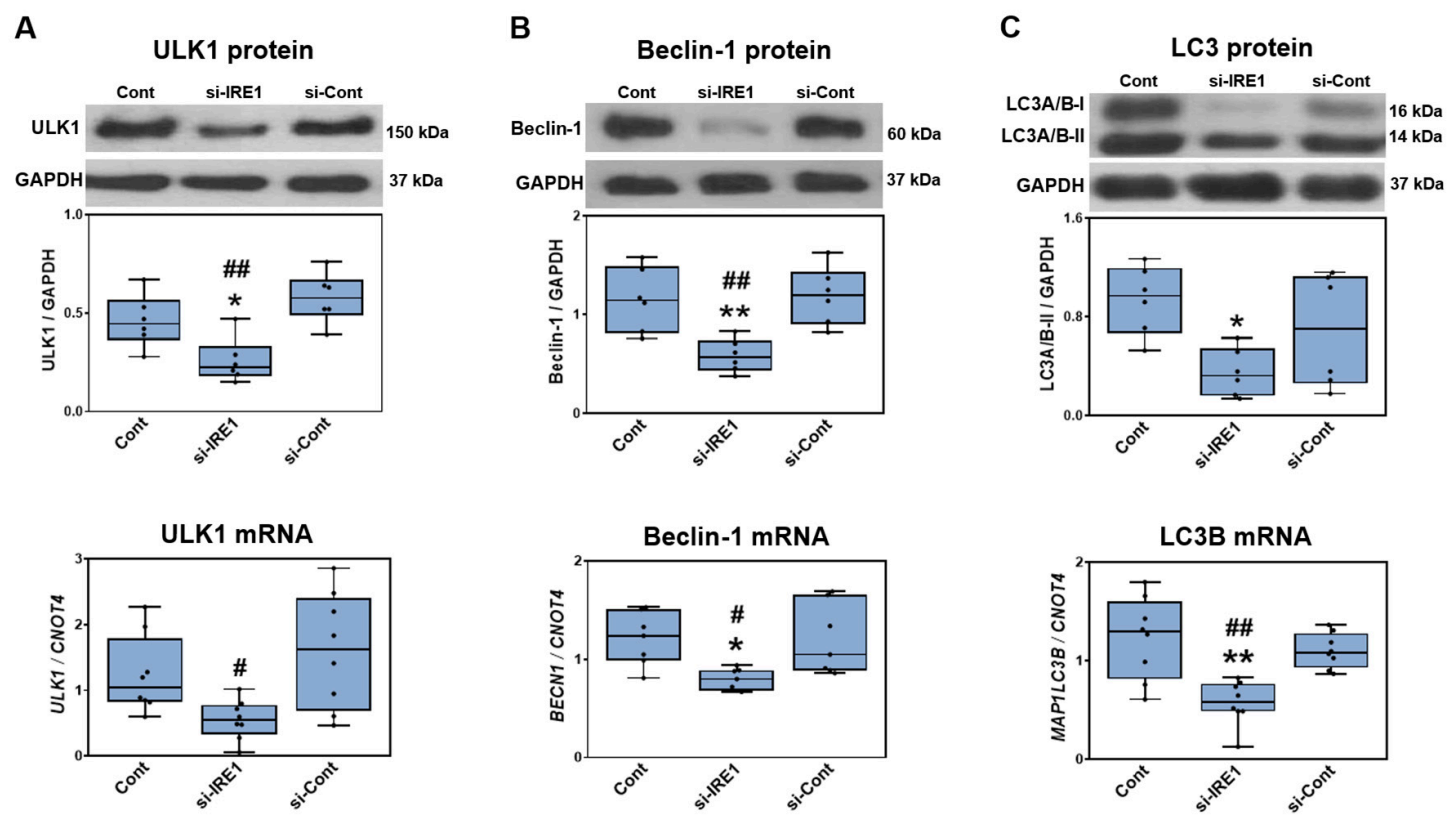
The effect of RNA interference of *ERN1* gene on the expression of autophagy genes and proteins in MOLT-3 cells. (**A**) Relative protein and mRNA content of ULK1. (**B**) Beclin-1 protein and gene expression. (**C**) Expression of LC3 at the translational and transcriptional levels. Shown are typical immunoblots and average data for protein content (n = 6, one-way ANOVA, Dunnett’s test) and gene expression (n = 8 for Kruskal–Wallis criterion, Dunn’s test). Cont—control without transfection, si-IRE1—RNA interference of *ERN1* gene, si-Cont—transfection with scrambled RNA. * *p* < 0.05, ** *p* < 0.01 compared to control; # *p* < 0.05, ## *p* < 0.01 compared to transfection with control siRNA.

Knockdown of *ERN1* in MOLT-3 cells suppressed the expression of genes encoding key markers of all major stages of autophagy—*ULK1* (Figure 7A), *BECN1* (Figure 7B), and *MAP1LC3B* (Figure 7C). A significant decrease in ULK1 and Beclin-1 protein levels was also observed. IRE1α depletion attenuated the expression of both cytoplasmic LC3A/B-I and autophagosome-bound LC3A/B-II proteins (Figure 7C). However, the level of lipidated LC3A/B-II protein declined only compared to the untreated control, whereas the difference between si-IRE1α- and si-Cont-treated cells was not statistically significant due to high variability of results. The LC3A/B-II/LC3A/B-I ratio—which indicates the conversion rate of LC3-I to LC3-II and is often used as an index of autophagy—was higher in IRE1α-silenced cells than in the untreated control, but not compared to cells transfected with scrambled siRNA. These findings indicate that IRE1α silencing affects the early stages of autophagy, suppressing the synthesis of autophagy effectors which may inhibit autophagic flux at the final stage.

## 3. Discussion

The present work, for the first time, established the distinct roles of PERK and IRE1α in inducing ER stress-related apoptosis or autophagy signaling in leukemic MOLT-3 cells. Knockdown of the *EIF2AK3* gene suppressed cell viability (Figure 1), upregulated both canonical and ER stress-related markers of apoptosis (caspase-3, Bax, and CHOP) (Figure 3), but did not affect the expression of key autophagy effectors (Figure 4). However, previous studies have revealed controversial effects of PERK loss, depending on the cell type and the nature of the stressor, with or without the involvement of autophagy. Our finding that PERK suppression promotes apoptosis aligns with the study by Vandewynckel et al. [21], which showed that pharmacological inhibition of PERK (but not IRE1α) under ER stress or hypoxia decreased the viability of hepatocellular carcinoma HepG2 cells and reduced the growth of mouse HCC xenografts. PERK silencing in human osteosarcoma MG63 cells enhanced ER stress and apoptosis but inhibited autophagy [22]. Moreover, PERK suppression compromised chemoresistance in various cancer models. For instance, PERK siRNA had no significant effect on glioblastoma PS-1 and U87MG cells when applied alone but greatly suppressed cell viability in combination with irradiation [23]. In tunicamycin-stressed human choriocarcinoma BeWo cells, PERK knockdown impaired the ability of melatonin to induce a PERK-dependent UPR and increased apoptosis [24]. In contrast, transient knockdown of PERK in liver L02 and HepG2 cells inhibited the PERK/ATF4/CHOP signaling cascade and apoptosis following sodium palmitate-triggered ER stress [25]. Treatment of breast cancer cells MCF-7 and ZR-75-1 with PERK siRNA or with the chemical PERK inhibitor GSK2656157 suppressed the pro-apoptotic PERK/CHOP/ATF4 pathway and attenuated apoptosis induced by the PI3K inhibitor VPS34-IN1 [26]. In our study, PERK silencing resulted in the stimulation of apoptotic effectors without affecting autophagy markers. These results might indicate that the PERK pathway is an important contributor to the survival of MOLT-3 cells under basal ER stress, while the loss of PERK activity may compromise their resistance to UPR-mediated apoptosis.

The responses of T-lymphoblastic leukemia cells to IRE1α silencing are more complicated and sometimes paradoxical. Thus, treatment of cells with si-IRE1α suppressed the translation of IRE1α protein but stimulated the transcription of the *ERN1* gene, suggesting a feedback mechanism to maintain IRE1α levels. Next, IRE1α loss did not produce any noticeable effect on the expression of the pro-apoptotic protein Bax, but suppressed caspase-3 and JNK (Figure 6) and reduced the expression of cellular markers of all major stages of autophagy (Figure 7). Partially similar to our findings, IRE1α knockdown prevented apoptosis and reduced the intracellular content of UPR-related (ATF6, GRP78, XBP1, CHOP) and apoptosis-related (PARP1, active caspase-3) proteins in osteosarcoma 143B and MG63 cells [27]. The transfection of IRE1α siRNA into gastric cancer cells inhibited autophagy caused by kaempferol [28]. However, IRE1α silencing did not change the level of ER stress-associated apoptosis of human nasopharyngeal carcinoma cells induced by trans-Resveratrol [29] and had no significant effect on apoptosis-related proteins in breast cancer cells following treatment with the PI3K kinase blocker VPS34-INI [26]. Pharmacological IRE1α inhibition increased the sensitivity of glioblastoma cells to temozolomide [30], while IRE1α knockdown slowed the growth of murine ES-2 ovarian cancer cell xenografts and increased the sensitivity of cells to cisplatin [31]. In our study, the suppression of major autophagy markers in MOLT-3 cells following IRE1α siRNA transfection suggests a direct link between the IRE1α UPR branch and autophagy signaling, while the inhibition of caspase-3 alongside autophagy effectors (Figure 6 and Figure 7) might indicate that apoptosis of these cells under basal conditions involves UPR-dependent autophagy. A close link between autophagy and apoptosis was shown in our previous study, in which the treatment of MOLT-3 cells with chloroquine increased the expression of both caspase-3 and LC3B genes, especially in serum-free medium [32]. The concurrent suppression of apoptotic (caspase-3, CHOP, JNK) and autophagic markers indicates that IRE1α silencing keeps cells in a quiescent state rather than promotes either death pathway. This suggests that IRE1α normally maintains a basal level of both processes, which may be required for cellular homeostasis.

The cause of such differential responses of MOLT-3 cells to PERK and IRE1α silencing might be attributed to their distinct functions. PERK regulates cellular stress by inhibiting global protein synthesis via eIF2α phosphorylation, selectively stimulating ATF4 and activating adaptive UPR genes as well as apoptosis/autophagy-associated genes such as CHOP and GADD34 [10]. IRE1α either restores ER homeostasis by splicing XBP1 mRNA into sXBP1 to increase protein folding, or initiates cell death by triggering RIDD-mediated degradation of specific mRNAs to diminish protein load [33]. The association between IRE1α and autophagy is also well established. The ability of the IRE1α/XBP1 pathway to transcriptionally regulate *BECN1* gene expression upon binding to its promoter provides a direct mechanistic link between UPR activation, autophagy, and cell fate decisions [34]. When IRE1α is silenced, reduced sXBP1 activity leads to decreased Beclin-1 expression, limiting both autophagosome formation and apoptotic signaling. This may explain why caspase-3 and CHOP are suppressed along with autophagy markers. Overall, the findings of our study might indicate that PERK loss inhibits the ability of cells to restore ER homeostasis, thus resulting in excessive ER stress and apoptosis. In contrast, IRE1α knockdown results in suppression of autophagy, which is closely linked with pro-apoptotic processes, although concurrent activation of these two types of cell death is also possible.

The cause of the stable expression of the anti-apoptotic Bcl-2 protein following the silencing of both PERK and IRE1α is unclear but might be attributed to the frequent upregulation of Bcl-2 in malignant cells, which contributes to apoptosis resistance [35]. Moreover, Bcl-2 mediates the interplay between autophagy and apoptosis [36]. The Beclin-1/Bcl-2 interaction inhibits autophagy, whereas dissociation of this complex due to competitive Bcl-2/Bax binding leads to the activation of autophagy. The stable expression of Bcl-2 in MOLT-3 cells observed in our study might indicate an attempt of the cells to stimulate protective autophagy.

The observation that IRE1α silencing decreases *EIF2AK3* gene expression (Figure 5C) is another intriguing finding of our study. The probable cause of this phenomenon is the existence of bidirectional communication between the PERK- and IRE1α-dependent UPR pathways. For instance, depletion of PERK with siRNA increased p-IRE1α levels in human ovarian carcinoma OVCAR-8 cells [19]. Using various knockdown genotypes in ER-stressed mouse fibroblasts, Tsuru et al. [37] established that the PERK/ATF4 branch enhances IRE1α/XBP1 signaling by increasing the efficiency of XBP1 splicing. Decreased expression of IRE1α and sXBP1 was observed in thapsigargin-stressed breast cancer MDA-MB-231 cells after pharmacological PERK inhibition [38]. Our study demonstrates the reverse direction—IRE1α appears to support PERK expression. This could occur through several mechanisms, for example, sXBP1 binding to the PERK promoter or regulation of transcription factors that control PERK expression. A similar effect was reported in the study by Ong et al. [38], where the application of IRE1α inhibitors (MKC8866, 4μ8c, and KIRA6) or IRE1α silencing reduced PERK protein content, suggesting that IRE1α signaling maintains PERK expression during chronic ER stress via binding of sXBP1 to the PERK promoter. Although the question of how the UPR switches between cytoprotective and pro-apoptotic mechanisms remains unresolved, it has been suggested that the key event is located between ATF4 and CHOP in the PERK cascade [39].

In conclusion, PERK functions primarily as a survival factor in MOLT-3 cells. PERK silencing removes a protective mechanism, allowing ER stress to reach a threshold that triggers unresolved ER stress and a cellular cascade involving CHOP induction, Bax upregulation, mitochondrial apoptosis, and cell death. The IRE1α pathway appears to be a stress surveillance mechanism that maintains both autophagic and apoptotic capacity. IRE1α knockdown suppresses both pathways but primarily activates non-mitochondrial apoptosis (the caspase-3/JNK/CHOP pathway) rather than Bax-mediated death. Our study also demonstrates crosstalk between the IRE1α and PERK cascades of the UPR, as components of the IRE1α pathway can influence the PERK branch. The differential consequences of PERK and IRE1α silencing on apoptotic and autophagic markers further highlight the diversity of regulatory mechanisms in tumor cells and underscore the importance of cell-specific targeting of the UPR in anticancer therapy.

From a therapeutic perspective, suppressing PERK activity appears to be a more promising approach than IRE1α inhibition for the elimination of leukemia cells when combined with conventional cytotoxic agents, as PERK silencing promotes apoptosis without stimulating protective autophagy. However, there are currently no PERK inhibitors approved for the treatment of leukemia. Highly selective PERK inhibitors GSK2606414 and GSK2656157, despite their antitumor activity in chronic lymphocytic leukemia [40] and acute myeloid leukemia [41] cells, exhibited a pronounced off-tumor side effect on pancreatic function in mice due to the inhibition of PERK activity in normal pancreatic cells [5,42]. In contrast, the observation that IRE1α knockdown reduces cell death and stimulates survival signaling suggests that IRE1α activation, rather than suppression, might be beneficial for anticancer therapy. However, no selective IRE1α agonists such as compounds IXA4/IXA62 [43] have yet advanced to clinical trials. On the other hand, a few IRE1α/XBP1 inhibitors have been examined in leukemia cell lines *in vitro*. For instance, sunitinib was tested in cultured myelomonocytic leukemia cells [44], while MKC8866 (ORIN1001) was evaluated in acute lymphoblastic leukemia [45].

In the absence of non-toxic pharmacological PERK and IRE1 modulators, RNA interference might be another powerful tool offering opportunities for combination anticancer therapy [16,17]. However, interdependence between UPR branches suggests that targeting one pathway may induce unintended compensatory upregulation of the other. Since UPR is essential in many normal tissues, long-term PERK and IRE1α modulation may have off-target effects. This complicates the development of UPR-targeted gene therapies. In addition, currently available siRNA preparations have low molecular stability, limited bioavailability, and a short half-life *in vivo* [16]. Nevertheless, several dozen drugs based on RNA interference technology are being tested in clinical trials. Gene therapy has demonstrated its considerable potential in experimental studies for the treatment of several types of cancer, including glioblastoma [46], pancreatic ductal adenocarcinoma [47], breast cancer [48], and thyroid cancer [49].

## 4. Materials and Methods

### 4.1. Cell Culture

T-lymphoblastic leukemia MOLT-3 cell line was ordered from Biolot (Saint-Petersburg, Russia). The cell suspensions were grown in RPMI-1640 medium supplemented with 4.5 g/L of glucose, 10% fetal bovine serum, 50 U/mL of penicillin, and 50 mg/mL of streptomycin (Biolot, Russia) at a temperature of 37 °C and 5% CO_2_.

### 4.2. RNA Interference

All reagents were obtained from Santa Cruz Biotechnology (Dallas, TX, USA): PERK siRNA (sc-36212), IRE1a siRNA (sc-40705), control siRNA (sc-37007), siRNA transfection reagent (sc-29528), siRNA transfection medium (sc-36868), buffer for siRNA dilution (sc-29527). RNA interference procedure was performed as recommended by the manufacturer (Santa Cruz Biotechnology, Dallas, TX, USA). The specificity and effectiveness of knocking down the target genes were confirmed using control siRNAs (a set of random siRNAs) as negative controls. The siRNA solutions and the transfection reagent were diluted separately in 100 µL of transfection medium, mixed at a 1:1 ratio, and further mixed with 800 µL of transfection medium. The aliquots of MOLT-3 cell suspensions were placed into a 6-well plate filled with transfection medium without serum and antibiotics at a density of ~3 × 10^5^ cells per well. The transfection mixture (final volume of 1 mL) contained either 10 µM of PERK siRNAs, IRE1 siRNAs, or control siRNAs.

Before studying the effects of PERK and IRE1α silencing on apoptosis and autophagy effectors, the optimal time of RNA interference was established. The expression of PERK and IRE1α at transcriptional and translational levels was compared after treatment of MOLT-3 cells with siRNA against PERK (si-PERK) and IRE1α (si-IRE1α) for 6 and 12 h at 37 °C and 95% O_2_/5% CO_2_. To confirm the absence of nonspecific effects of siRNA transfection, the cells were also cultured under control conditions (untreated cells) and incubated with control siRNA (si-Cont). After treatment, the transfection mixture was supplemented with 1 mL of standard RPMI-1640 medium containing 20% fetal bovine serum and antibiotics, and the cells were grown for 24 h. Finally, the medium was replaced with complete RPMI-1640 medium, and the cells were cultured for another 48 h. The cells were transfected in parallel for RT-PCR and immunoblotting analyses.

As shown in Figure S1A, 6 h transfection of si-PERK led to considerable suppression of both PERK protein and *EIF2AK3* gene in comparison to control and si-Cont. The expression levels of PERK gene and protein in untreated cells and the cells cultured with scrambled siRNA were similar. However, increasing the transfection time to 12 h resulted in a pronounced upregulation of PERK in the cells exposed to control siRNA in comparison to untreated cells, although this difference reached statistical significance only at the transcriptional level. Taking into account such nonspecific effect of si-Cont on MOLT-3 cells after longer treatment, further RNA interference experiments were performed for 6 h.

The analysis of IRE1α knockdown efficiency in dependence on the time yielded unexpected results (Figure S1B). Thus, the transfection of si-IRE1α led to a pronounced decrease in IRE1α protein content after both 6 h and 12 h exposure. However, 6 h transfection was accompanied by a significant increase in the relative expression of *ERN1* gene, while after 12 h incubation the IRE1α mRNA content was comparable in the cells treated with si-IRE1α or si-Cont, and in untreated cells. Taking into account such an interesting phenomenon observed after 6 h, but an absence of effect following 12 h, further si-IRE1α transfections were performed for 6 h as well.

### 4.3. MTT Assay

The viability of cells was evaluated by MTT assay as described earlier [32]. The intensity of the colorimetric reaction was determined using the plate reader CLARIOstar Plus (BMG Labtech, Ortenberg, Germany) at a wavelength of 570 nm. The percentage of viable cells treated with siRNAs was calculated by comparing their extinction with that of the cells incubated under control conditions and taken as 100%.

### 4.4. RT-PCR Analysis

MOLT-3 cells were collected and lysed with a single-phase aqueous ExtractRNA reagent (Eurogen, Moscow, Russia) in the proportion of 1 mL per 10^6^ of cells. The resulting lysates were centrifuged at 12,000× *g* and 4 °C for 10 min. To isolate RNA, the supernatants were collected and mixed with chloroform (0.2 mL per 1 mL of ExtractRNA solution), and the mixture was centrifuged for 15 min at 12,000× *g* and 4 °C. The aqueous phase containing RNA was aspirated, mixed with isopropanol (0.5 mL per 1 mL of ExtractRNA solution), and centrifuged for 10 min at 12,000× *g* and 4 °C. The resulting precipitate was dissolved in 75% ethanol and stored at −20 °C for subsequent analysis. Total RNA content in the samples was determined by the ratio of absorption coefficients at 260/280 nm using a NanoPhotometer-N50 spectrophotometer (IMPLEN, Munich, Germany).

To synthesize cDNA, 1 µg of RNA from the samples was mixed with 1 µL of Random(dN)10-primer dN reagent (Eurogen, Moscow, Russia). The reverse transcription was performed using 4 µL of Storage Buffer 5× (Eurogen, Moscow, Russia), 2 µL of dNTP, 2 µL of DTT, 2 µL of dH_2_O and 1 µL of MMLV revertase from the MMLV RT kit (Eurogen, Moscow, Russia). The reaction mixture was sequentially heated to 25 °C for 5 min, to 42 °C for 60 min, and to 70 °C for 5 min. The synthesized cDNA was diluted 10-fold with dH_2_O and stored at −20 °C. PCR reactions were performed on a C1000 Touch Thermal Cycler equipped with a CFX96 detection unit (Bio-Rad Laboratories, Inc., Hercules, CA, USA). The reaction mixture contained 1 µL of synthesized cDNA, 1 µL (10 nM) of forward and reverse primers, 17 µL of dH_2_O and 5 µL of qPCRmix-HS SYBR Master Mix reagent (Evrogen, Moscow, Russia). The amplification scheme included initial denaturation (95 °C for 5 min) and 45 cycles of direct amplification (denaturation at 95 °C for 5 s, annealing at 57–63 °C for 10–50 s, and elongation at 72 °C for 30 s). All amplification reactions were performed in triplicate. All the genes were also analyzed without a DNA template. The results of PCR reactions were processed using CFX Manager software 3.0 (Bio-Rad Laboratories, Inc., Hercules, CA, USA). The specificity of amplification was verified by one peak on the melting curves. 

All the primers used in the work were synthesized by Evrogen (Moscow, Russia) and exhibited high amplification efficiency (close to ideal value of 2) and a correlation coefficient of 0.98 or above. The melting points have been optimized using Primer Blast software (https://www.ncbi.nlm.nih.gov/tools/primer-blast/ (accessed on 12 July 2026)). The sequences of primers used in the work are shown in Table 1. The relative expression of target genes was assessed by 2^−ΔΔCT^ method and normalized to the expression of reference gene CNOT4 (subunit 4 of the CCR4-NON-transcription complex) [50]. The results of RT-PCR analysis were expressed in relative units; the average expression level in the control samples was taken as one.

### 4.5. Immunoblotting

The cells were lysed with a cold RIPA buffer system (Santa Cruz Biotechnology, USA) containing a mixture of cellular protease and phosphatase inhibitors, and left on ice for 15 min. Cell lysates were frozen and stored at a temperature of −80 °C until analysis. The total protein content in the samples was determined by the Lowry method.

The sample proteins (30 µg per well) were denatured and separated by molecular weight by SDS-PAGE electrophoresis using 7.5%, 10% or 12% acrylamide gels depending on the molecular weight of the studied proteins. Then the proteins were transferred to a nitrocellulose membrane (Amersham GE HealthCare, Buckinghamshire, UK), whose non-specific binding was blocked for 1 h with a 5% solution of skimmed milk dissolved in TTBS buffer (Tris-HCl and 0.1% Tween-20). The membranes were consequently treated with the primary antibodies against target proteins (Table 2) and the secondary anti-rabbit antibodies conjugated with horseradish peroxidase (1:2000). Immunopositive signals of the proteins were visualized on X-ray film using an ECL chemiluminescent solution (Cytiva, Marlborough, MA, USA). To control the protein loading, the membranes were stripped and treated with the primary mouse antibodies to GAPDH. The relative expression of the proteins was evaluated by densitometric analysis using Image J program 1.54 (NIH, Bethesda, MD, USA). The optical density of the target proteins was normalized to the density of GAPDH bands.

### 4.6. Statistical Analysis

The results were analyzed using GraphPad Prism 8.1 software (San Diego, CA, USA). First, the data were checked for outliers (using the ROUT method with a value of α = 0.05) and the normality of the distribution (Shapiro–Wilk criterion). In the case of normal distribution, the statistical significance was assessed by one-way ANOVA for multiple comparisons followed by Dunnet’s test. If the results failed the normality test, the nonparametric Kruskal–Wallis criterion for multiple comparisons and the post hoc Dunn’s test were used. The differences were considered statistically significant at *p* < 0.05.

## 5. Limitations and Perspectives

The authors acknowledge a few limitations of the study. First, while MOLT-3 cells represent a well-established T-lymphoblastic leukemia model, the findings may not generalize to all leukemia subtypes or solid tumors. Second, these findings need to be verified *in vivo*. Next, given that ER stress responses are highly dynamic, the 72 h post-transfection observation period provides insight into the chronic effects of gene silencing but may miss early adaptive responses. It is known that ER stress stimulates all UPR cascades, often simultaneously triggering antagonistic (protective and pro-death) mechanisms, while the functioning of individual UPR arms depends on the duration of ER stress, with PERK showing early activation and IRE1α demonstrating more sustained signaling [51]. For instance, after the rapid initial activation of the UPR, PERK signaling contributes to cell death under persistent ER stress, whereas IRE1 signaling is attenuated under such conditions. Therefore, the cell’s fate after silencing of UPR effectors might greatly depend on post-transfection time. Thus, shorter time points following PERK and IRE1α RNA interference in MOLT-3 cells could be an interesting goal for future studies aiming to reveal additional mechanistic details. Finally, our work was limited by evaluating the expression of only a few key apoptosis and autophagy markers. However, the cellular network connecting ER stress, apoptosis, and autophagy in cancers includes multiple interacting signaling pathways, and studying the expression of other proteins that might function as switches between survival and death of cancer cells is important for the development of new targets for clinical oncology. Suppressing PERK activity in combination with cytotoxic agents in both *in vitro* and *in vivo* experiments is also required to further evaluate the effectiveness of this approach for the clinical treatment of T-lymphoblastic leukemia.

## Figures and Tables

**Table 1:** Nucleotide sequences, temperature, and annealing time of primers used for PCR analysis.

Gene	Gene Sequence (NCBI)	Forward Primer (5′ → 3′) Reverse Primer (3′ → 5′)	Annealing Temperature (°C) and Time (s)
*EIF2AK3*	NM_004836.7	Forward: TGTCGCCAATGGGATAGTGACGAA Reverse: AATCCGGCTCTCGTTTCCATGTCT	59, 20
*ERN1*	NM_001433.5	Forward: CAGCAGACTTTGTCATCGGC Reverse: CTCTCGGGTTTTGGTGTCGT	59, 30
*BAX*	NM_001291429.2	Forward: TCCCCCCGAGAGGTCTTTT Reverse: CGGCCCCAGTTGAAGTTG	57, 10
*BCL2*	NM_000633.3	Forward: AGTACCTGAACCGGCACCT Reverse: GGCCGTACAGTTCCACAAA	57, 50
*CASP3*	NM_032991.2	Forward: AACTGGACTGTGGCATTGAG Reverse: ACAAAGCGACTGGATGAACC	56, 30
*ULK1*	NM_003565.4	Forward: GGCAAGTTCGAGTTCTCCCG Reverse: CGACCTCCAAATCGTGCTTCT	59, 50
*BECN1*	NM_001313998.2	Forward: CCATGCAGGTGAGCTTCGT Reverse: GAATCTGCGAGAGACACCATC	57, 50
*MAP1LC3B*	NM_022818.5	Forward: TTCAGGTTCACAAAACCCGC Reverse: TCTCACACAGCCCGTTTACC	57, 30
*CNOT4* *	NM_001393371.1	Forward: GTCCAAAACCTGACTGCATGTATC Reverse: GGTGTTTACCCGCCTGCAT	57, 10

*—Reference gene.

**Table 2:** Primary antibodies used in the study.

Target Protein	Antibody Type	Dilution	Manufacturer	Catalogue Number
PERK	a/rabbit	1:1000	Abcam (Waltham, MA, USA)	Ab229912
p-eIF2α	a/rabbit	1:500	Abcam	Ab32157
IRE1α	a/rabbit	1:500	AbClonal (Woburn, MA, USA)	A1731
sXBP1	a/rabbit	1:1000	Cell Signaling (Danvers, MA, USA)	83418
GRP78	a/rabbit	1:500	ServiceBio (Wuhan, China)	GB11098
Bax	a/rabbit	1:1000	Cell Signaling	2772
Bcl-2	a/rabbit	1:500	Cell Signaling	2764
Caspase-3	a/rabbit	1:500	Cell Signaling	14220
Cleaved Caspase-3	a/rabbit	1:200	Cell Signaling	9664
CHOP	a/rabbit	1:500	ServiceBio	GB115691
JNK1/2/3	a/rabbit	1:1000	AbClonal	A18678
ULK1	a/rabbit	1:200	Cell Signaling	8054
Beclin-1	a/rabbit	1:500	Cell Signaling	3495
LC3A/B	a/rabbit	1:500	ServiceBio	GB11124
GAPDH	a/mice	1:1000	Santa Cruz	sc-166545

## Data Availability

The original contributions presented in this study are included in the article/Supplementary Materials. Further inquiries can be directed to the corresponding author.

## Supplementary Materials

The following supporting information can be downloaded at: https://www.mdpi.com/article/10.3390/ijms27156588/s1.
