# Supplementary material for: Differential Effects of PERK and IRE1α Silencing on Expression of Apoptosis and Autophagy Markers in T-Lymphoblastic Leukemia MOLT-3 Cells
Source: Int J Mol Sci. 2026 Jul 24;27(15):6588. doi: 10.3390/ijms27156588 (PMC13465333; doi:10.3390/ijms27156588)
Supplement: Supplementary file 1 [file ijms-27-06588-s001.zip › ijms-4362271-supplementary.pdf]

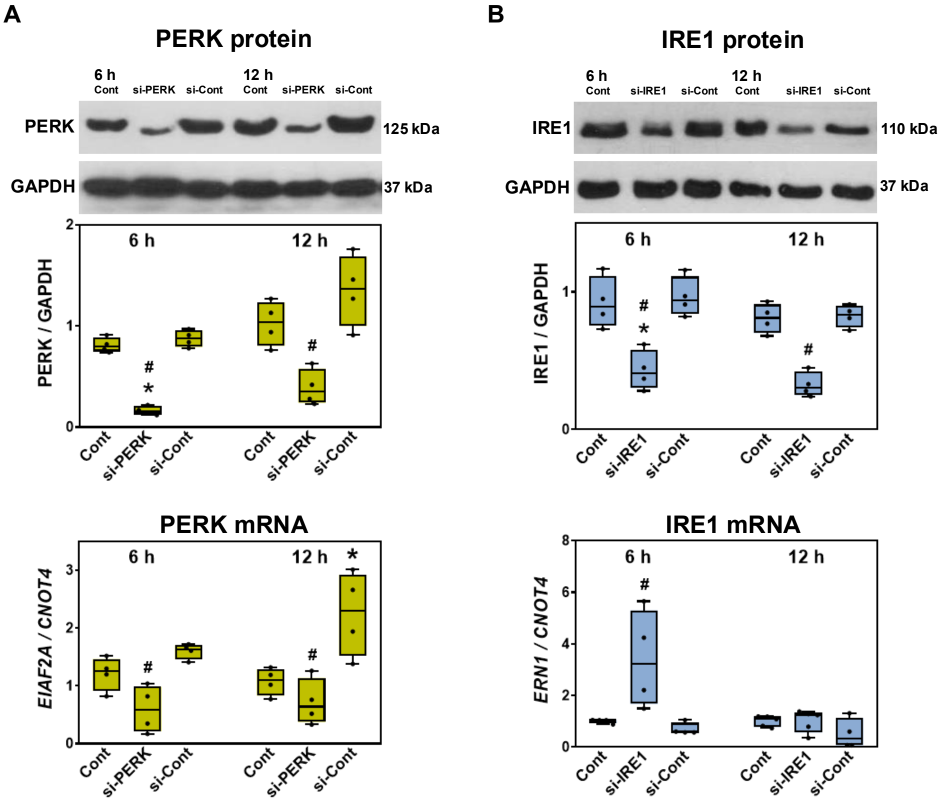

Figure S1. The dependence of PERK (**A**) and IRE1α (**B**) protein and gene expression on the time of siRNA transfection. Cont – control without transfection, si-PERK – RNA interference of *EIAF2AK3* gene, si-IRE1 – RNA interference of *ERN1* gene, si-Cont – transfection with control RNA. * $p < 0.05$ in comparison to control; # $p < 0.05$ compared to transfection with control siRNAs (n=4, Kruskal-Wallis criterion for multiple comparisons with Dunn test).
